# High expression of PPFIA1 in human esophageal squamous cell carcinoma correlates with tumor metastasis and poor prognosis

**DOI:** 10.1186/s12885-023-10872-9

**Published:** 2023-05-09

**Authors:** Yongyin Gao, Lizhao Guan, Ran Jia, Wanyi Xiao, Youming Han, Yue Li, Peng Tang, Zhentao Yu, Hongdian Zhang

**Affiliations:** 1 Tianjin Medical University Cancer Institute and Hospital, National Clinical Research Center for Cancer, Tianjin’s Clinical Research Center for Cancer, Key Laboratory of Cancer Prevention and Therapy of Tianjin, Tianjin, 300060 China; 2 Department of Biochemistry and Molecular Biology, School of Basic Medical Sciences, Tianjin Medical University, Tianjin, 300070 China; 3 National Cancer Center, National Clinical Research Center for Cancer, Cancer Hospital & Shenzhen Hospital, Chinese Academy of Medical Sciences and PeKing Union Medical College, Shenzhen, 518116 China; 4 Binhai Hospital of Tianjin Medical University General Hospital, Tianjin, 300456 China

**Keywords:** Esophageal squamous cell carcinoma, PPFIA1, Bioinformatics analysis, Prognosis, Migration and invasion

## Abstract

**Background:**

PTPRF interacting protein alpha 1 (PPFIA1) is reportedly related to the occurrence and progression of several kinds of malignancies. However, its role in esophageal squamous cell carcinoma (ESCC) is unclear. This current study investigated the prognostic significance and biological functions of PPFIA1 in ESCC.

**Methods:**

Oncomine, Gene Expression Profiling Interactive Analysis (GEPIA), and Gene Expression Omnibus (GEO) were used to investigate *PPFIA1* expression in esophageal cancer. The relationship between PPFIA1 expression and clinicopathological characteristics and patient survival was evaluated in GSE53625 dataset, and verified in the cDNA array based on qRT–PCR and tissue microarray (TMA) dataset based on immunohistochemistry. The impact of PPFIA1 on the migration and invasion of cancer cells were investigated by wound-healing and transwell assays, respectively.

**Results:**

The expression of PPFIA1 was obviously increased in ESCC tissues versus adjacent esophageal tissues according to online database analyses (all *P* < 0.05). High PPFIA1 expression was closely related to several clinicopathological characteristics, including tumor location, histological grade, tumor invasion depth, lymph node metastasis, and tumor-node-metastasis (TNM) stage. High PPFIA1 expression was related to worse outcomes and was identified as an independent prognostic factor of overall survival in ESCC patients (GSE53625 dataset, *P* = 0.019; cDNA array dataset, *P* < 0.001; TMA dataset, *P* = 0.039). Downregulation of PPFIA1 expression can significantly reduce the migration and invasion ability of ESCC cells.

**Conclusion:**

PPFIA1 is related to the migration and invasion of ESCC cells, and can be used as a potential biomarker to evaluate the prognosis of ESCC patients.

**Supplementary Information:**

The online version contains supplementary material available at 10.1186/s12885-023-10872-9.

## Background

Esophageal cancer is ranked as the eighth most common cancer and the sixth leading cause of cancer-related death worldwide [1]. Esophageal squamous cell carcinoma (ESCC) is the main pathological subtype, accounting for nearly 90% of esophageal cancer tumors in China [2]. Surgical resection and neoadjuvant or adjuvant therapy are the main methods for ESCC treatment. Despite advances in diagnosis and therapy, recurrence and metastasis are common and significantly decrease the overall survival (OS) of patients [3]. Therefore, there is an urgent need to identify the metastatic mechanisms of ESCC and to explore effective indicators for early diagnosis, prognosis prediction, and treatment response evaluation.

PTPRF interacting protein alpha 1 (PPFIA1) is a member of the LAR protein-tyrosine phosphatase-interacting protein family of cytosolic scaffold proteins [4]. Several studies have reported that PPFIA1 is abnormally expressed, plays a vital role in invasion and metastasis, and may be used as a potential prognostic molecular marker for several types of malignancies, including breast cancer, laryngeal cancer, oral squamous cell carcinoma, colon cancer and ovarian cancer [5–10]. However, the expression of PPFIA1 and its correlation with clinicopathological features and patient prognosis in ESCC remain uncertain.

In the present research, we first investigated the expression of PPFIA1 in ESCC tissues and paracancerous tissues using available online datasets from the Oncomine, Gene Expression Profiling Interactive Analysis (GEPIA), and Gene Expression Omnibus (GEO) databases. The associations of PPFIA1 expression with the clinicopathological features and outcomes of ESCC patients were then analyzed in the GSE53625 dataset and further confirmed in cDNA array and tissue microarray (TMA) datasets. Finally, the effects of PPFIA1 on the migration and invasion of ESCC cells were investigated.

## Materials and methods

### GEPIA

The expression of *PPFIA1* mRNA in human tumors was analyzed through the GEPIA website (http://gepia.cancer-pku.cn/detail.php), which contains RNA sequencing and expression data from The Cancer Genome Atlas (TCGA) and the Genotype-Tissue Expression (GTEx) databases [11].

### Oncomine

Oncomine (http://www.oncomine.org) is a public online microarray database and comprehensive data mining platform that can be used to mine cancer genetic information [12].

### GEO

The GEO database (https://www.ncbi.nlm.nih.gov/geo/) is a publicly accessible database of gene expression data that stores a large amount of microarray data [13]. The GEO datasets were analyzed online through GEO2R.

Furthermore, the RNA sequencing data, all clinicopathological variable data and the survival data of 179 ESCC patients were also downloaded from the GSE53625 dataset (https://www.ncbi.nlm.nih.gov/geo/query/acc.cgi?acc=GSE53625). There were 146 (81.6%) males and 33 (18.4%) females, with a median age of 60 (range, 36 ~ 82) years. According to the criteria of the seventh edition of the American Joint Committee on Cancer (AJCC) tumor-node-metastasis (TNM) staging system for ESCC, 10 (5.6%), 77 (43.0%), and 92 (51.4%) cases were considered stage I, II, and III, respectively.

### TCGA

The RNA-sequencing data and clinical information of 81 ESCC patients and 11 normal esophageal samples were downloaded from TCGA database (https://cancergenome.nih.gov/). The level of gene expression was measured as fragments per kilobase of transcript per million mapped reads (FPKM) [14].

### Kaplan–Meier analysis

Kaplan–Meier Plotter (www.kmplot.com) is an online database containing survival information (including recurrence-free survival [RFS], progression-free survival [PFS], time to first progression [FP], OS, postprogression survival [PPS], and distant metastasis-free survival [DMFS]) for patients with breast, ovarian, lung or gastric cancers. The cancer samples were divided into two groups (low vs. high *PPFIA1* expression) based on the automatically selected best cutoff values.

### Search Tool for the Retrieval of Interacting Genes (STRING)

We further used the STRING website (http://string-db.org/) to explore the interactions between PPFIA1 and other genes based on a generated protein–protein interaction (PPI) network [15].

### Analysis of tissue cDNA array data based on quantitative real-time polymerase chain reaction (qRT-PCR)

An ESCC cDNA microarray with patient diagnosis information was purchased from Shanghai Outdo Biotech Co., Ltd. (Cat. No.: cDNA-HEsoS095Su01; Shanghai, China); the array contained samples from 67 cancer tissues and 28 adjacent esophageal tissues. These tissues were all from patients who were histologically confirmed to have ESCC and underwent esophagectomy. The tissues were from 48 males and 19 females, with a median age of 59 (range, 37 ~ 78) years. The study was approved by the Medical Ethics Committee of the Shanghai Outdo Biotech Company.

Total RNA was isolated using TRIzol reagent (Takara, Dalian, China) and the cDNA was then synthesized using the SuperScript™ II Reverse Transcriptase kit (Thermo Fisher Scientific, Waltham, MA, USA) according to the manufacturer’s instructions. Then, 2 µl cDNA was used as a template for qRT–PCR with SYBR® Premix Ex Taq™ II (Takara, Dalian, China), and β-actin was used as the internal control. The primer sequences used were as follows: PPFIA1, forward 5'-CTTAACCCAGGGGAAGTTACAC-3' and reverse 5'-ATCCTAAGAGACCGCTCATGC-3'; and β-actin, forward 5'-GAAGAGCTACGAGCTGCCTGA-3' and reverse 5'-CAGACAGCACTGTGTTGGCG-3'. The expression fold-changes were quantified by the 2^−∆∆CT^ method.

### Immunohistochemistry (IHC) analysis

TMAs containing samples from 147 ESCC tissues and 40 adjacent normal esophageal tissues were made by Shanghai Outdo Biotech Co. Ltd. All paraffin block specimens were obtained from patients who underwent radical esophagectomy between 2009 and 2010 at Tianjin Medical University Cancer Institute and Hospital, with reliable information on survival. No patients had received any chemotherapy or radiotherapy before surgery. Among the patients, there were 119 males and 28 females with a median age of 68 years old. All patients were followed up until September 2016 with a median follow-up period of 36 months. The research protocol was approved by the Research Ethics Committee of Tianjin Medical University Cancer Institute and Hospital, and written informed consent was obtained from all patients.

The protein expression of PPFIA1 was detected through IHC. In brief, the tissue sections were deparaffinized, rehydrated and incubated with an antigen retrieval solution. The activity of endogenous peroxidase was blocked with 0.3% H_2_O_2_ and 5% goat serum. Then, the sections were incubated with an anti-PPFIA1 polyclonal antibody (1:100; Cat No. DF12102) at 4 °C overnight and then incubated with a biotinylated secondary antibody for 20 min at room temperature. Diaminobenzidine was used as a chromogen. The stained slide was scanned using an automatic slice scanning system.

Two or three experienced pathologists who were blinded to the clinicopathological information independently performed the staining evaluation based on the staining intensity (no staining, 0; weak, 1; moderate, 2; strong, 3) and the percentage (0%, 0; 1% ~ 25% positive, 1; 26% ~ 50% positive, 2; 51% ~ 75% positive, 3; 76% ~ 100% positive, 4) of positively stained tumor cells. The immunoreactivity score was calculated as the product of the staining intensity and staining percentage scores, with the final score ranging from 0 to 12. Patients with a total score of < 4 were considered to have low expression, and those with a total score of ≥ 4 were considered to have high expression.

### Cell culture and transfection

The human ESCC cell lines Kyse-30 and Ec-109 were grown in RPMI 1640 medium containing 10% fetal bovine serum (Gibco, USA). The siRNA targeting PPFIA1 (si-PPFIA1) and negative control were synthesized by GenePharma (Shanghai, China). ESCC cells were transiently transfected with these siRNAs using Lipofectamine 2000 reagent (Invitrogen, Life Technologies) following the manufacturer's instructions. 48 h after transfection, the RNA and protein were harvested and analyzed.

### Western blotting

Western blotting was performed as described previously [16]. Total protein was extracted using RIPA buffer supplemented with protease inhibitors. The protein concentration was detected by a BCA Reagent Kit. Equal amount of protein was electrophoresed and transferred onto PVDF membranes. Non-specific binding was blocked with 5% skim milk. The membranes were then incubated with primary antibodies overnight at 4 °C and secondary antibodies at room temperature for 2 h. Protein expression was visualized with an enhanced chemiluminescence system.

### Cell migration and invasion assays

The wound-healing assay was conducted to evaluate cellular migration according to our published protocol [17]. ESCC cells were seeded into 6-well plates. When the cell confluence achieved about 80 ~ 90%, scratches were created by scraping the cell layer using a 10 μl pipette tips across each well. After washing with PBS, the cells were cultured with serum-free medium at 37 °C containing 5% CO2. The remaining cells were cultured for another 48 h.

Cells which suspended with serum-free medium were plated into the upper chamber of an insert Transwell chambers (Corning, NY, USA) with or without coated Matrigel (BD Biosciences, CA, USA). The lower chambers were filled with medium containing 10% FBS. After incubation for 36 h at 37 °C, migrated or invasive cells on the lower surface of the membrane were fixed and stained with crystal violet, and cells in five randomly selected fields were photographed and counted.

### Statistical analyses

Statistical analyses were performed using SPSS 17.0 software (SPSS, Inc., Chicago, IL) and GraphPad Prism 7.0 software (GraphPad, Inc., La Jolla, CA, USA). Data were expressed as the mean ± standard deviation (SD). The differences between the groups were assessed using Student's t test. The patients were divided into two groups according to the best cutoff value of PPFIA1 expression for predicting OS calculated with the X-tile 3.6.1 software (Yale University, New Haven, CT, USA) for analysis of the GSE53625 dataset [18] or according to the median value of PPFIA1 expression for analysis of the cDNA array dataset. The chi-square test or Fisher’s exact test was used for the analysis of PPFIA1 expression and clinicopathological variables. Survival analysis was carried out through the Kaplan–Meier method and the log-rank test. The correlations between PPFIA1 expression and the expression of other genes were assessed by Pearson’s correlation analysis. A *P* value < 0.05 was deemed statistically significant.

## Results

### The expression of *PPFIA1* mRNA in various tumor tissues

**Fig. 1:**
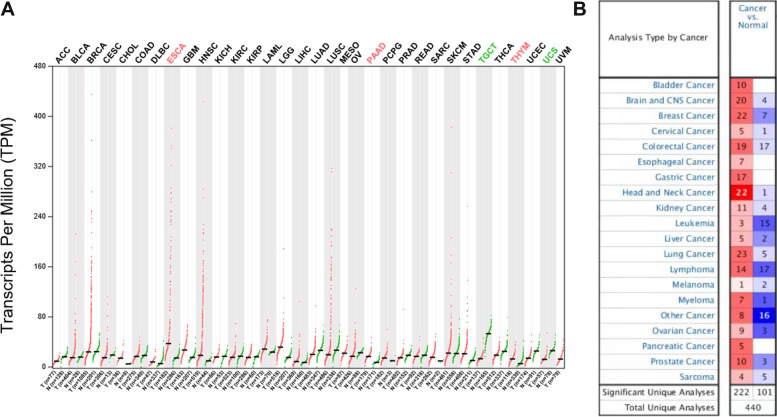
Overview of *PPFIA1* mRNA expression. **A** The PPFIA1 expression profile of tumor tissues and paired normal tissues was analyzed using the GEPIA website. **B** *PPFIA1* mRNA expression in different tumor tissues compared with normal controls was analyzed using the Oncomine database

We first examined the expression level of *PPFIA1* mRNA in various tumors with the GEPIA website, and the results showed significantly higher *PPFIA1* mRNA expression in several types of tumor tissues, including esophageal cancer (ESCA), pancreatic adenocarcinoma (PAAD), and thymoma tissues, compared with adjacent normal tissues (Fig. 1A). Overall PPFIA1 expression in various tumor specimens, as well as in normal controls, were further analyzed with the Oncomine database. *PPFIA1* mRNA expression was obviously increased in the tumor tissues versus normal tissues from most of the datasets for all types of tumors (Fig. 1B). The fold changes in *PPFIA1* mRNA expression in different ESCA tissues of 7 datasets are displayed in Supplementary Table 1.

### The expression of PPFIA1 was upregulated in ESCC tissues

**Fig. 2:**
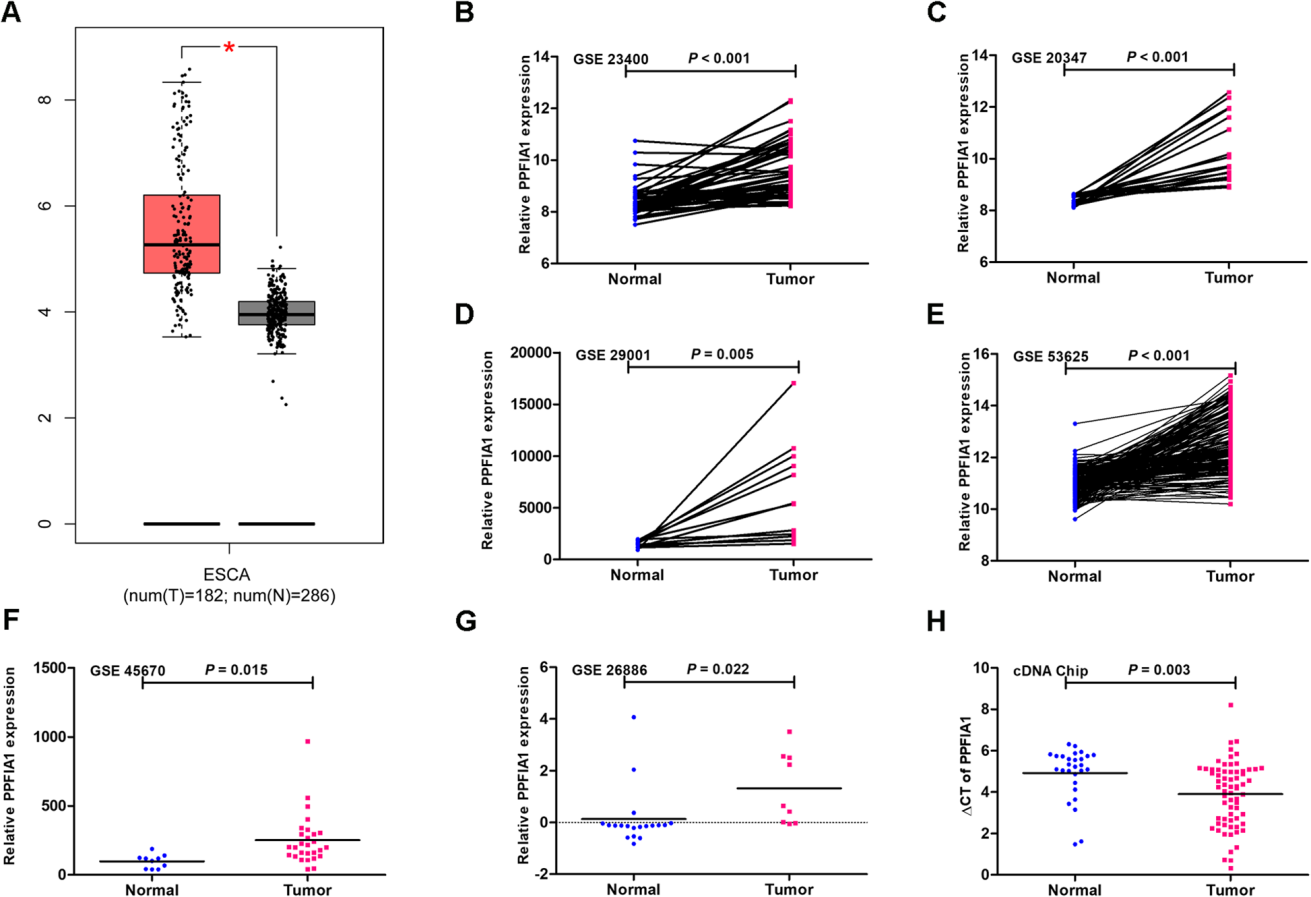
The expression of *PPFIA1* mRNA in ESCC patients. **A** *PPFIA1* mRNA expression in ESCC tissues versus normal tissues was analyzed using GEPIA datasets. *PPFIA1* mRNA was highly expressed in ESCC tissues compared to noncancerous tissues in the GSE23400 (**B**), GSE20347 (**C**), GSE29001 (**D**), GSE53625 (**E**), GSE45670 (**F**) and GSE26886 (**G**) datasets. **H** *PPFIA1* mRNA expression was determined in 67 tumor tissues and 28 normal tissues (cDNA array) using qRT–PCR analysis. *P < 0.05 comparison between normal and tumor tissues

The expression of *PPFIA1* mRNA was markedly higher in ESCA tumor samples than in adjacent normal samples according to the GEPIA website (Fig. 2A). This finding was further verified using data from six GEO datasets; the results indicated that the relative *PPFIA1* mRNA expression was obviously upregulated in ESCC tissues compared with paired or unpaired adjacent normal tissues in the GSE23400 (9.6 ± 1.1 vs. 8.4 ± 0.6, *P* < 0.001, Fig. 2B), GSE20347 (10.3 ± 1.3 vs. 8.4 ± 0.2, *P* < 0.001, Fig. 2C), GSE29001 (6395.6 ± 4748.9 vs. 1498.6 ± 337.9, *P* = 0.005, Fig. 2D), GSE53625 (12.6 ± 1.2 vs. 11.0 ± 0.5, *P* < 0.001, Fig. 2E), GSE45670 (251.5 ± 185.9 vs. 97.5 ± 49.6, *P* = 0.015, Fig. 2F) and GSE26886 (1.3 ± 1.4 vs. 0.1 ± 1.1, *P* = 0.022, Fig. 2G) datasets.

In addition, the increased expression of *PPFIA1* mRNA in ESCC samples was further confirmed through the analysis of cDNA array data based on qRT–PCR (*P* = 0.003, Fig. 2H).

### Correlations between *PPFIA1* mRNA expression and clinicopathological variables in ESCC

In the GSE53625 dataset, the 179 ESCC patients were divided into a low-expression group (*n* = 155) and a high-expression group (*n* = 24) according to the X-tile cutoff. Our results showed that high PPFIA1 expression was positively related to tumor invasion depth (*P* = 0.019), lymph node metastasis (*P* = 0.013), and TNM stage (*P* = 0.012), but no correlations were found between PPFIA1 expression and gender, age, smoking use, alcohol use, tumor location, histological grade or adjuvant therapy (*P* > 0.05) (Table 1).

**Table 1:** Associations of *PPFIA1* mRNA expression with clinicopathological variables of 179 ESCC patients from the GSE53625 dataset

Clinicopathological variables	Number	PPFIA1 expression		*x*^*2*^	*P* value
		Low	High		
Gender				0.649	0.420
Male	146	125	21		
Female	33	30	3		
Age (years)				0.316	0.574
≤ 60	99	87	12		
˃60	80	68	12		
Smoking use				2.872	0.090
None	65	60	5		
Yes	114	95	19		
Alcohol use				0.637	0.425
None	73	65	8		
Yes	106	90	16		
Tumor location				0.771	0.680
Upper	20	16	4		
Middle	97	85	12		
Lower	62	54	8		
Histological grade				1.557	0.459
I	32	29	3		
II	98	86	12		
III	49	40	9		
Tumor invasion depth				7.935	**0.019**
T1-T2	39	38	1		
T3	110	94	16		
T4a	30	23	7		
Lymph node metastasis				10.840	**0.013**
N0	83	76	7		
N1	62	56	6		
N2	22	15	7		
N3	12	8	4		
TNM stage				8.878	**0.012**
I	10	10	0		
II	77	72	5		
III	92	73	19		
Adjuvant therapy				1.768	0.184
None	45	42	3		
Yes	104	87	17		
unknown	30				

*Abbreviations*: *PPFIA1* PTPRF interacting protein alpha 1, *ESCC* Esophageal squamous cell carcinoma, *TNM* tumor-node-metastasis

We then analyzed PPFIA1 levels in the cDNA array data of 67 ESCC patients. The patients were grouped into a low-expression group (*n* = 34) and a high-expression group (*n* = 33) based on the median value of relative PPFIA1 expression. We found that *PPFIA1* mRNA expression was significantly correlated with histological grade (*P* = 0.031, Supplementary Table 2).

### Correlations between PPFIA1 protein expression and clinicopathological variables in ESCC patients in the TMA dataset

**Fig. 3:**
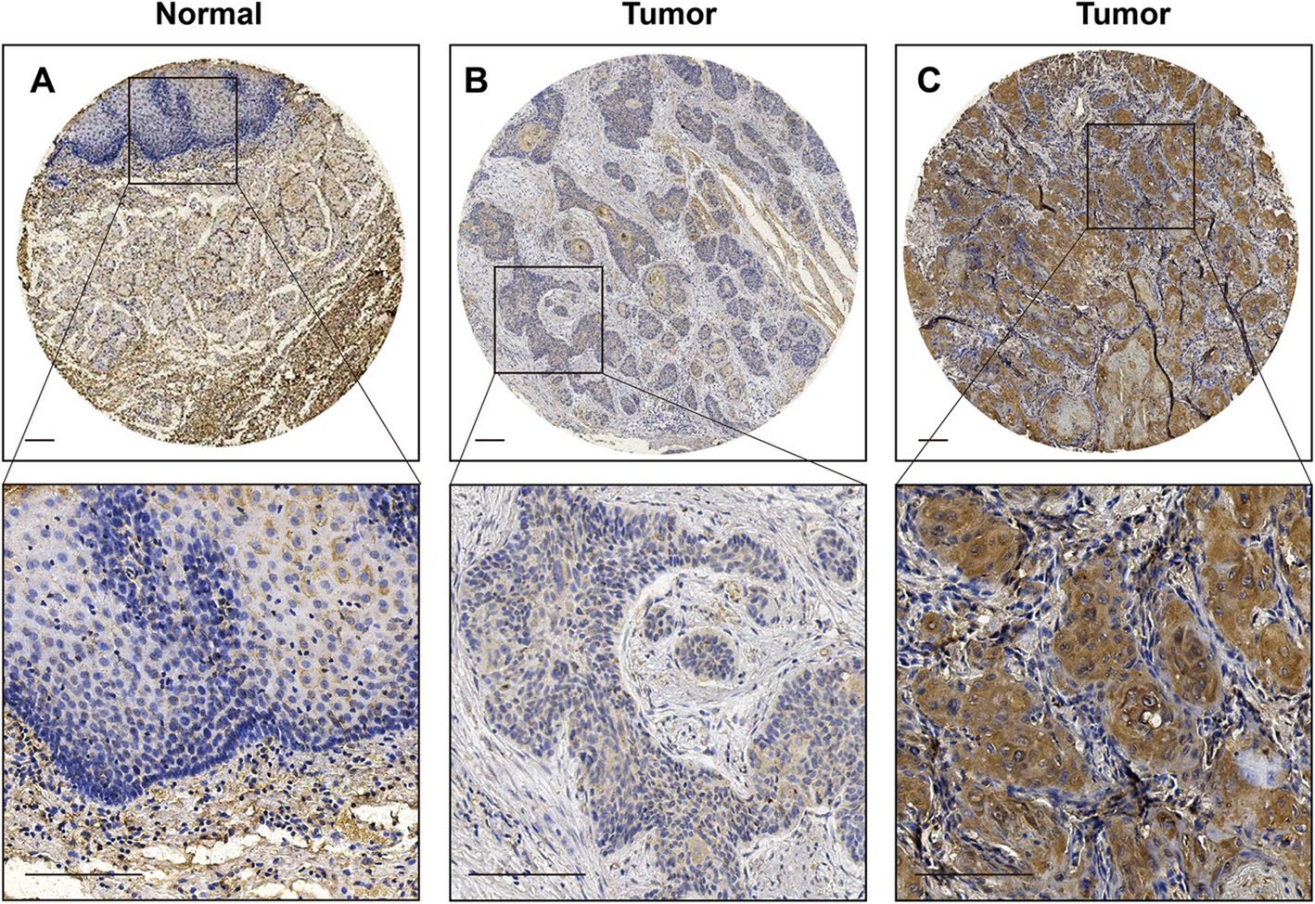
The protein expression of PPFIA1 in ESCC tissues was detected in a tissue microarray. **A** Negative PPFIA1 expression in sections of nonneoplastic mucosa adjacent to tumors. **B** Low PPFIA1 expression in ESCC tissue. **C** High PPFIA1 expression in ESCC tissue

We further assessed PPFIA1 protein expression using an IHC staining-based TMA dataset containing samples from 147 surgically removed cancer tissues and 40 normal esophageal tissues. PPFIA1 was primarily localized to the cytoplasm and nucleus of cancer cells, and the expression rate was significantly higher in cancer tissues than in normal tissues (68.0% vs. 25.0%, *P* < 0.05). Representative images of low or high PPFIA1 expression are shown in Fig. 3A-C. All patients were divided into the PPFIA1 low-expressing group (*n* = 47) or the high-expressing group (*n* = 100). Correlation analysis found that PPFIA1 expression was inversely associated with tumor location (*P* = 0.011), tumor invasion depth (*P* = 0.041), lymph node metastasis (*P* = 0.020), and TNM stage (*P* = 0.007) in ESCC patients (Table 2).

**Table 2:** Correlations between PPFIA1 protein expression and clinicopathological variables in 147 patients with ESCC from the TMA dataset

Clinicopathological variables	Number	PPFIA1 expression		*x*^*2*^	*P* value
		Low	High		
Gender				< 0.001	0.983
Male	119	38 (31.9%)	81 (68.1%)		
Female	28	9 (32.1%)	19 (67.9%)		
Age(years)				1.433	0.231
≤ 68	77	28 (36.4%)	49 (63.6%)		
˃68	70	19 (27.1%)	51 (72.9%)		
Smoking use				3.280	0.070
None	77	20 (26.0%)	57 (74.0%)		
Yes	70	28 (40.0%)	42 (60.0%)		
Tumor location				9.067	**0.011**
Upper	6	0 (0)	6 (100.0%)		
Middle	123	37 (30.1%)	86 (69.9%)		
Lower	18	10 (55.6%)	8 (44.4%)		
Tumor size (cm)				0.803	0.370
< 3.5	61	22 (36.1%)	39 (63.9%)		
≥ 3.5	86	25 (29.1%)	61 (70.9%)		
Histological grade				1.565	0.457
I	11	5 (45.5%)	6 (54.5%)		
II	115	37 (32.2%)	78 (67.8%)		
III	21	5 (23.8%)	16 (76.2%)		
Tumor invasion depth				6.369	**0.041**
T1- T2	22	11 (50.0%)	11 (50.0%)		
T3	80	27 (33.8%)	53 (66.3%)		
T4	45	9 (20.0%)	36 (80.0%)		
Lymph node metastasis				5.449	**0.020**
None	86	34 (39.5%)	52 (60.5%)		
Yes	61	13 (21.3%)	48 (78.7%)		
TNM stage				7.344	**0.007**
I/II	55	25 (45.5%)	30 (54.5%)		
III	92	22 (23.9%)	70 (76.1%)		

*Abbreviations*: *PPFIA1* PTPRF interacting protein alpha 1, *ESCC* esophageal squamous cell carcinoma, *TNM* tumor-node-metastasis, *TMA* TMA, tissue microarray

### Increased PPFIA1 expression indicates a poor prognosis for malignancies

In view of the high expression of *PPFIA1* mRNA in a variety of malignancies, including breast, ovarian, lung, and gastric cancers, we further investigated the prognostic value of PPFIA1 through the Kaplan–Meier Plotter website. Patients were grouped based on the automatically selected best cutoff value for PPFIA1 expression. As shown in Supplementary Fig. 1, the expression of PPFIA1 apparently correlated with the RFS, OS, PPS, and DMFS of patients with breast cancer (*P* < 0.05); the PFS, OS, and PPS of patients with ovarian cancer (*P* < 0.05); the FP, OS, and PPS of patients with lung cancer (*P* < 0.05); and the FP, OS, and PPS of patients with gastric cancer (*P* < 0.05). These results suggest that high expression of PPFIA1 is obviously associated with worse outcomes in patients with breast cancer, ovarian cancer, lung cancer, and gastric cancer.

### Correlations between PPFIA1 expression and the prognosis of ESCC patients

**Fig. 4:**
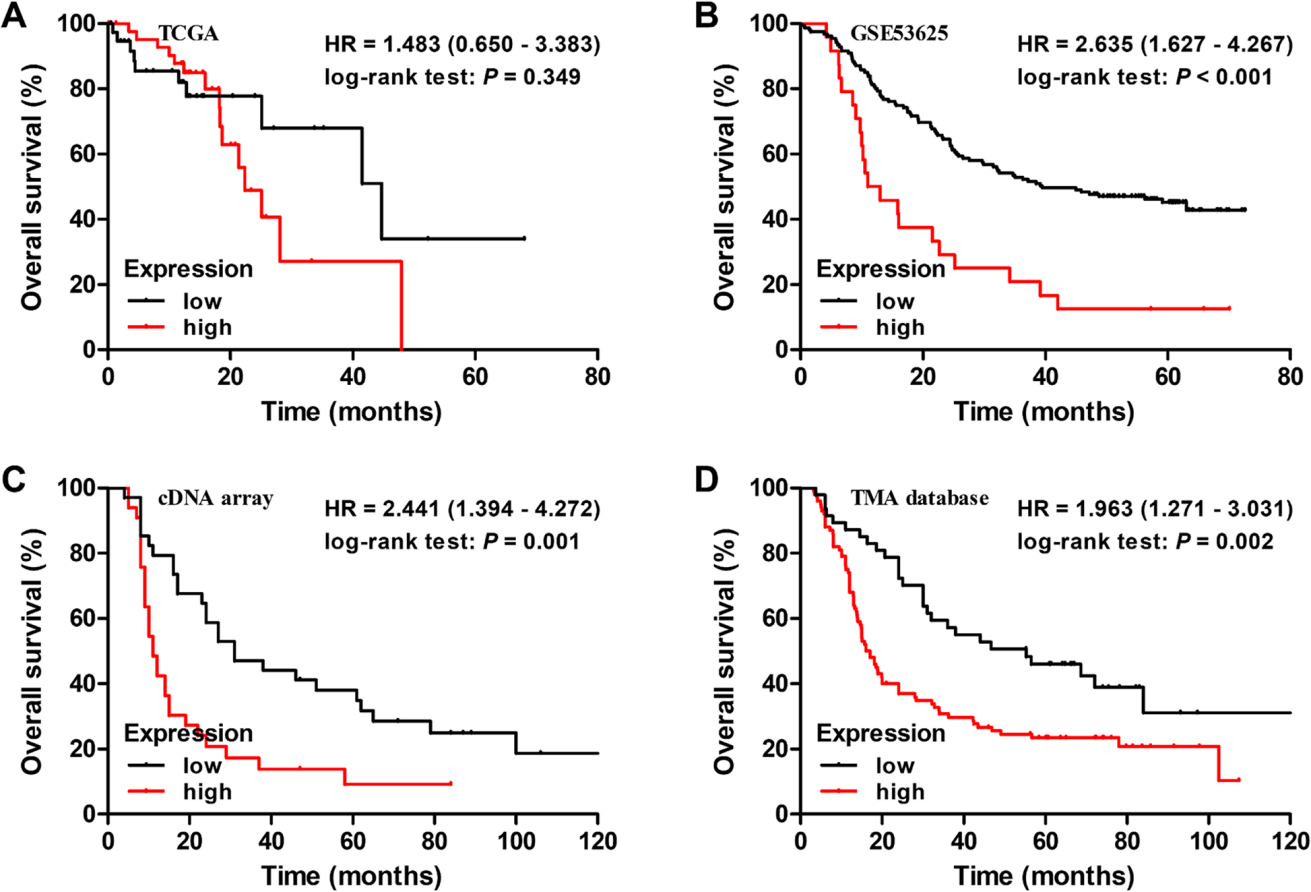
The association of PPFIA1 expression with the OS of ESCC patients. **A** Kaplan‒Meier curve of the survival of patients stratified by PPFIA1 mRNA expression in the TCGA dataset. **B** Kaplan‒Meier curve of the survival of patients stratified by *PPFIA1* mRNA expression in the GSE53625 dataset. **C** Kaplan‒Meier curve of the survival of patients stratified by PPFIA1 mRNA expression in the cDNA array dataset. **D** Kaplan‒Meier curve of PPFIA1 protein expression stratification in the TMA dataset

Survival analyses revealed that the median survival time of ESCC patients with high and low PPFIA1 expression were similar (22.7 months vs. 45.37 months, *P* = 0.349, Fig. 4A) in the TCGA database. ESCC patients with high *PPFIA1* mRNA levels had a significantly worse 5-year OS rate than those with low *PPFIA1* mRNA levels in the GSE53625 dataset (12.5% vs. 45.2%, *P* < 0.001, Fig. 4B) and cDNA array dataset (9.2% vs. 38.0%, *P* = 0.001, Fig. 4C). High PPFIA1 expression at the protein level was also obviously related to poor outcomes in patients in the TMA dataset (5-year OS rate: 23.4% vs. 46.0%, *P* = 0.002, Fig. 4D).

**Fig. 5:**
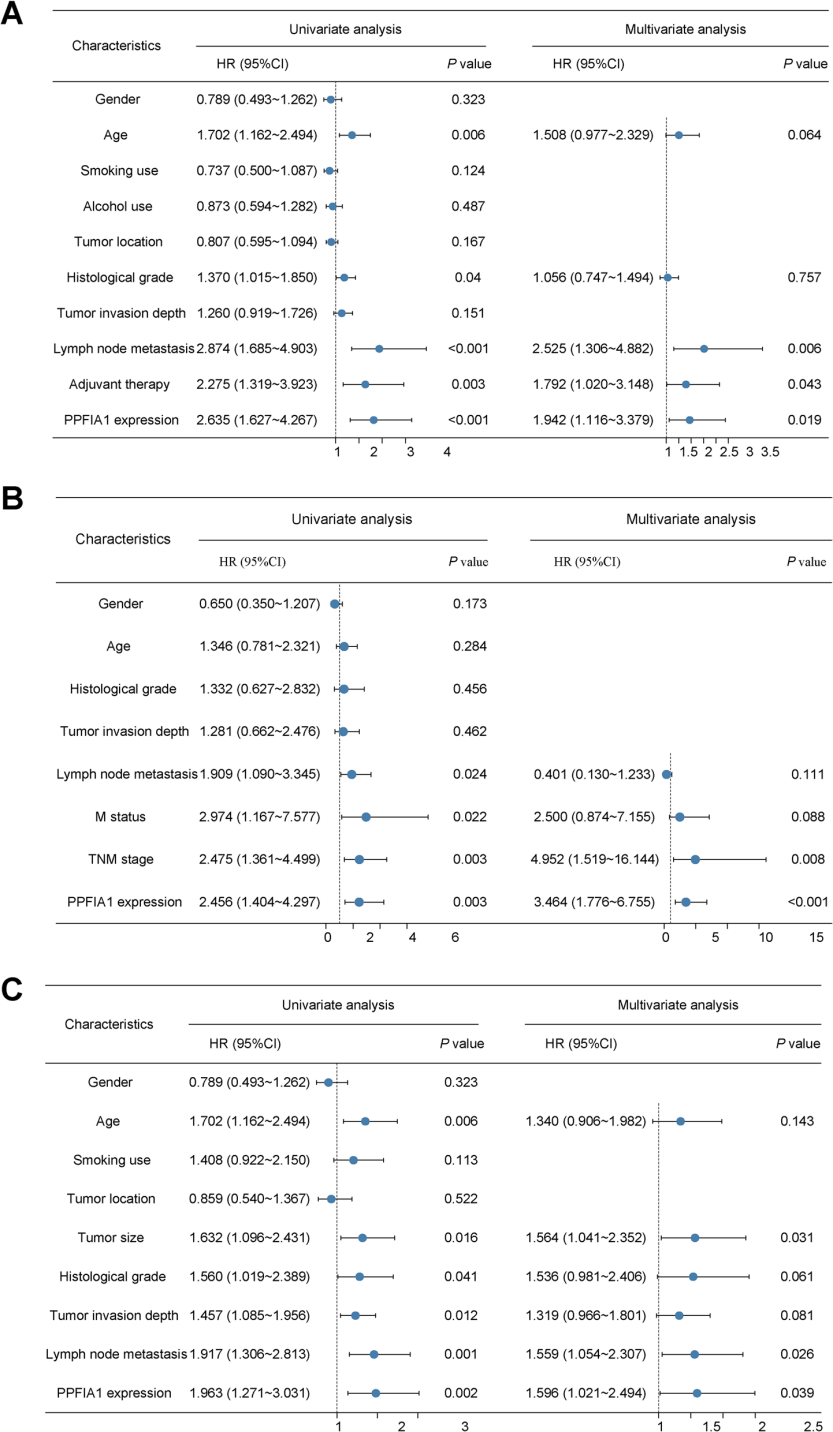
Univariate and multivariate analyses of factors affecting the overall survival of ESCC patients in the GSE53625 (**A**), cDNA array (**B**) and TMA (**C**) datasets

The prognosis-related indicators in the 3 datasets were analyzed by univariate and multivariate survival analyses. In the GSE53625 dataset, age, histological grade, lymph node metastasis, adjuvant therapy and PPFIA1 expression were significantly related to OS. In the cDNA array dataset, lymph node metastasis, M status, TNM stage and PPFIA1 expression were related to OS. Moreover, age, tumor size, histological grade, tumor invasion depth, lymph node metastasis, and PPFIA1 expression were significantly related to OS in the TMA dataset. Furthermore, PPFIA1 was identified as an independent risk factor for poor OS in the multivariate Cox proportional hazard regression analysis of all 3 datasets (GSE53625 dataset, HR = 1.942, *P* = 0.019, Fig. 5A; cDNA array dataset, HR = 3.464, *P* < 0.001, Fig. 5B; TMA dataset, HR = 1.596, *P* = 0.039, Fig. 5C). Other independent factors included tumor size (TMA dataset), lymph node metastasis (GSE53625 and TMA datasets), TNM stage (cDNA array dataset) and adjuvant therapy (GSE53625 dataset).

### The effect of PPFIA1 on the migration and invasion of ESCC cells in vitro

**Fig. 6:**
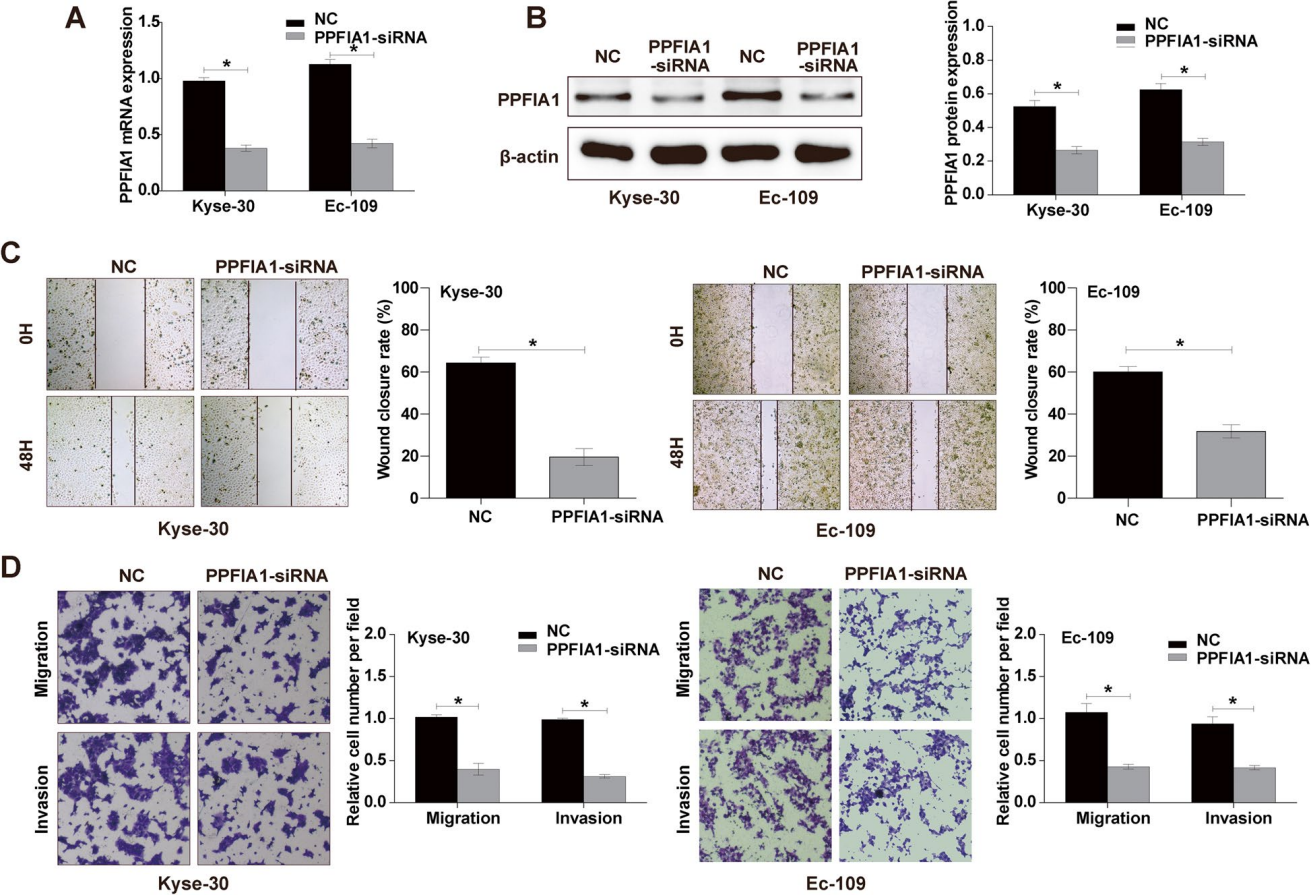
Effects of PPFIA1 knockdown on the migration and invasion of ESCC cells in vitro*.* The expression of PPFIA1 in Kyse-30 and Ec-109 cells after transfection with PPFIA1-siRNA was determined by qRT‒PCR (**A**) and western blotting (**B**), respectively. **C** Cell migration of ESCC cells was assessed by wound-healing assay. **D** Cell migration and invasion of ESCC cells were assessed by transwell assays. *P < 0.05

To further determine the role of PPFIA1 in ESCC cell migration and invasion, we transfected Kyse-30 and Ec-109 cells with PPFIA1-siRNA or negative control. We found the PPFIA1 expression at the mRNA (Fig. 6A) and protein (Fig. 6B) levels were downregulated by transfection with PPFIA1-siRNA (*P* < 0.05). The wound-healing assay showed that PPFIA1 knockdown significantly inhibited the migration ability of Kyse-30 and Ec-109 cells (Fig. 6C, *P* < 0.05). Furthermore, transwell assays showed that the relative migration and invasion abilities of Kyse-30 and Ec-109 cells were remarkably decreased after PPFIA1 knockdown (Fig. 6D, *P* < 0.05). Thus, these results indicate that the expression of PPFIA1 is crucial for the migration and invasion of ESCC cells.

### Network analysis of PPFIA1 and interacting genes

**Fig. 7:**
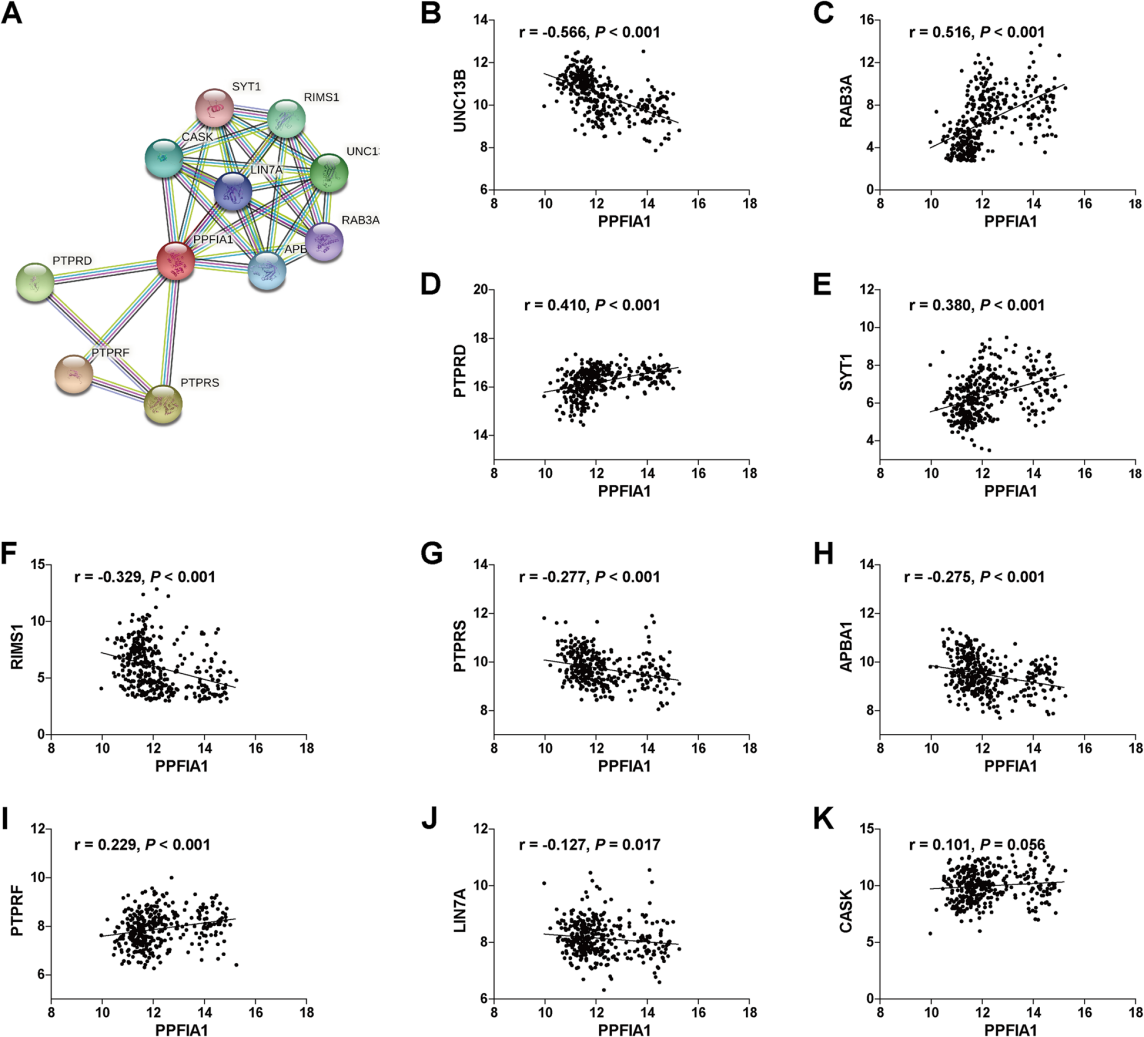
PPI network analysis of PPFIA1-related proteins. **A** Annotation of the genes that were associated with PPFIA1 using the STRING database. The correlations of PPFIA1 expression with UNC13B (**B**), RAB3A (**C**), PTPRD (**D**), SYT1 (**E**), RIMS1 (**F**), PTPRS (**G**), APBA1 (**H**), PTPRF (**I**), LIN7A (**J**), and CASK (**K**) expression were assessed by Pearson’s correlation analysis in the GSE53625 dataset

The interactions between PPFIA1 and other genes in the PPI network were analyzed via the STRING website. The results showed some known and predicted interactions between PPFIA1 and UNC13B, RAB3A, PTPRD, SYT1, RIMS1, PTPRS, APBA1, PTPRF, LIN7A, and CASK (Fig. 7A). Most of these genes have been reported as oncogenes or tumor suppressor genes.

We further analyzed the associations of *PPFIA1* mRNA expression with the abovementioned genes in the GSE53625 dataset. Our results showed that PPFIA1 expression was clearly correlated with UNC13B, RAB3A, PTPRD, SYT1, RIMS1, PTPRS, APBA1, PTPRF, and LIN7A expression (Fig. 7B-J) but not with CASK expression (Fig. 7K).

## Discussion

Early invasion and metastasis are two of the main reasons for the poor prognosis of patients with ESCC [19]. In our current study, we observed an increase of PPFIA1 expression in ESCC, which was associated with tumor metastasis and a poorer prognosis. PPFIA1 may be a potential molecular indicator for diagnosis and prognosis evaluation and could be regarded as a new therapeutic target for ESCC.

*PPFIA1* is a gene located at the 11q13 amplification region and mainly encodes the liprin-α1 protein in humans. It was initially discovered to control the formation and function of synapses in neurons [20]. Studies have indicated that PPFIA1 is a key regulator of cell motility, focal adhesion, cell signal transduction, and cytoskeletal organization [21, 22]. PPFIA1 is frequently amplified and plays a crucial role in cell migration and invasion by affecting cell motility, mediating extracellular matrix degradation and facilitating the formation of lamellipodial protrusions of cancer cells [5, 23, 24]. Although the role of PPFIA1 in tumor cell progression has been well verified, it is not clear whether its dysregulation is related to metastasis risk or prognosis in ESCC patients.

To determine the clinical value of PPFIA1 in ESCC, we analyzed the difference in the expression of this novel marker in tumor tissues compared with normal tissues and assessed its correlation with the survival of ESCC patients using multiple datasets. Analysis of datasets from Oncomine, GEPIA and GEO confirmed that *PPFIA1* mRNA expression was markedly higher in ESCC tissues than in adjacent normal control tissues, which was further confirmed with cDNA array data based on qRT–PCR and TMA data based on IHC. These results indicated that PPFIA1 may play a vital role during the tumorigenesis of ESCC, which is consistent with the results discovered in other malignant tumors [20, 23]. Further correlation analysis revealed that PPFIA1 expression is highly correlated with aggressive biological behaviors of tumors, indicating the important function of PPFIA1 in the progression of ESCC. It is worth mentioning that the expression of PPFIA1 was only correlated with the degree of tumor differentiation in the cDNA chip dataset, which might be due to the limited sample size in the research.

We further discovered that PPFIA1 can be applied for the prognostic assessment of breast cancer, ovarian cancer, lung cancer and gastric cancer by online Kaplan–Meier Plotter. Cho et al. [25]. explored the prognostic value of PPFIA1 alone or in combination with TMEM16A and FADD in patients with invasive breast cancer and found that combined expression was significantly associated with perineural invasion and a low disease-free survival rate. A recent study showed that the expression of PPFIA1 is closely related to poor response to endocrine treatment in luminal breast cancer [21]. Since PPFIA1 was found to be involved in the development of ESCC, it could be inferred that PPFIA1 is likely to have a great impact on the survival of patients with ESCC. To assess this possibility, we used the GSE53625, cDNA array and TMA datasets to investigate the prognostic value of PPFIA1 in ESCC. Our results indicated that high PPFIA1 expression was evidently correlated with a poorer prognosis. Notably, PPFIA1 was identified as an independent indicator of poor prognosis in all three independent databases in the multivariate analyses. Thus, PPFIA1 may serve as a potential diagnostic and novel prognostic biomarker, as well as a new therapeutic target for ESCC.

Since the expression of PPFIA1 is related to malignant biological indicators and poor prognosis, we further carried out cytological experiments to clarify the effect of PPFIA1 on the metastasis ability of ESCC cells. Our study found that down-regulation of PPFIA1 expression can significantly reduce the migration and invasion behavior of cancer cells. These data further support that PPFIA1 functions as an oncogene in the metastasis of ESCC.

Mechanistically, Shen et al. [26] confirmed that PPFIA1 enhances the proliferation and migration ability of colon carcinoma cells by interacting with the tumor suppressor protein ING4. Astro et al. [27] suggested that liprin-α1 can determine the polarization and morphological dynamics related to cell migration by forming a complex with the liprin-β1, ERC1/ELKS, and LL5 proteins. To examine the potential mechanisms of PPFIA1 in ESCC progression, we further used PPI network and Pearson’s correlation analyses to identify proteins that may bind to PPFIA1. We identified UNC13B, RAB3A, PTPRD, SYT1 and RIMS1 as potential candidates. Some of these interacting genes have already been reported as tumor oncogenes or suppressor genes. For instance, RAB3A interacting protein (Rab3IP) is a Rab-specific GEF, and the activation of Rab proteins, including RAB3A and RAB8, has been considered a tumor-specific marker in colorectal cancer, gastric cancer, and pancreatic cancer [28, 29]. A recent study reported by Ren et al. [30] showed that RAB3IP interacts with SSX2 and enhances the invasive aggressive phenotype of gastric cancer through epithelial-mesenchymal transition. PTPRT is a phosphatase that can participate in JAK/STAT signal transduction. Deleterious mutations or copy number loss of PTPRT and its related gene *PTPRD* are potential markers for evaluating resistance to bevacizumab regimens and are closely associated with shorter PFS in metastatic colorectal cancer patients [31]. However, the biological function and underlying mechanism of PPFIA1 in ESCC need to be further studied.

There are several certain limitations. First, although the results were analyzed through bioinformatics analysis and three independent databases (GSE53625, cDNA array and TMA), the sample size involved in the present study was relatively small. Second, the clinicopathological information from the three datasets was not comprehensive, and part of the information in the cDNA array dataset was incomplete. Third, the detailed molecular mechanism and signaling pathways by which PPFIA1 influences the migration and invasion of ESCC were not verified. Therefore, our results need to be further verified.

In conclusion, this study indicates that the expression of PPFIA1 is significantly increased and is related to migration and invasion behaviors, and poor outcomes in ESCC patients. PPFIA1 might be a valuable biomarker for early detection, treatment formulation and prognostic evaluation for ESCC.

## Supplementary Information

**Additional file 1:** **Supplementary Table 1.** Oncomine analysis of PPFIA1 mRNA expression in esophageal cancer.

**Additional file 2:** **Supplementary Table 2.** Correlations between PPFIA1 mRNA expression and clinicopathological variables in 67 ESCC patients in the cDNA array dataset.

**Additional file 3:** **Supplementary Fig. 1.** The correlations between PPFIA1 expression and the prognoses of patients with malignancies were analyzed using Kaplan-Meier Plotter.

**Additional file 4:** **Supplementary Fig. 2.** The full-length images of western blots in Fig. 6B.

## Data Availability

The datasets that support the findings of this study are available from third parties, with the weblinks provided in the manuscript methodology section and can be located at the following sites: GEPIA (http://gepia.cancer-pku.cn/detail.php), Oncomine (http://www.oncomine.org), GEO database (https://www.ncbi.nlm.nih.gov/geo/), GSE53625 (https://www.ncbi.nlm.nih.gov/geo/query/acc.cgi?acc=GSE53625). TCGA database (https://cancergenome.nih.gov/), Kaplan–Meier Plotter (www.kmplot.com) and STRING (http://string-db.org/).

## Abbreviations

ESCC: Esophageal squamous cell carcinoma
PPFIA1: PTPRF interacting protein alpha 1
GEPIA: Gene Expression Profiling Interactive Analysis
TMA: Tissue microarray
PPI: Protein–protein interaction
STRING: Search Tool for the Retrieval of Interacting Genes
RFS: Recurrence-free survival
PFS: Progression-free survival
FP: Time to first progression
OS: Overall survival
PPS: Post-progression survival
DMFS: Distant metastasis-free survival
