# Supplementary material for: High expression of PPFIA1 in human esophageal squamous cell carcinoma correlates with tumor metastasis and poor prognosis
Source: BMC Cancer. 2023 May 9;23:417. doi: 10.1186/s12885-023-10872-9 (PMC10169376; doi:10.1186/s12885-023-10872-9)
Supplement: Supplementary file 1 — Additional file 1: Supplementary Table 1. Oncomine analysis of PPFIA1 mRNA expression in esophageal cancer. [file 12885_2023_10872_MOESM1_ESM.pdf]

Supplementary Table 1 Oncomine analysis of *PPFIA1* mRNA expression in esophageal cancer

Cohort No.	Cohort	Data type	Sample (n)	Fold change	*P* value
1	Hu Esophagus (34)	mRNA	Esophageal squamous cell carcinoma (17) vs. normal (17)	3.745	7.60E-06
2	Su Esophagus 2 (106)	mRNA	Esophageal squamous cell carcinoma (53) vs. normal (53)	2.257	4.14E-10
3	Wang Esophagus (52)	mRNA	Barrett's esophagus (19) vs. normal (24)	1.268	3.20E-02
4	Hu Esophagus 2 (132)	DNA	Esophageal squamous cell carcinoma (30) vs. blood (102)	1.845	5.11E-06
5	Kimchi Esophagus (24)	mRNA	Esophageal adenocarcinoma (8) vs. normal (8)	2.091	1.60E-02
6	Kim Esophagus (118)	mRNA	Esophageal adenocarcinoma (75) vs. normal (28)	1.337	7.75E-06
			Barrett's esophagus (15) vs. normal (28)	1.344	1.00E-03

Abbreviations: PPFIA1, PTPRF interacting protein alpha 1
