# Supplementary material for: High expression of PPFIA1 in human esophageal squamous cell carcinoma correlates with tumor metastasis and poor prognosis
Source: BMC Cancer. 2023 May 9;23:417. doi: 10.1186/s12885-023-10872-9 (PMC10169376; doi:10.1186/s12885-023-10872-9)
Supplement: Supplementary file 2 — Additional file 2: Supplementary Table 2. Correlations between PPFIA1 mRNA expression and clinicopathological variables in 67 ESCC patients in the cDNA array dataset. [file 12885_2023_10872_MOESM2_ESM.pdf]

Supplementary Table 2 Correlations between *PPFIA1* mRNA expression and clinicopathological variables in 67 ESCC patients in the cDNA array dataset

Clinicopathological variables	Number	PPFIA1 expression		x^2	*P* value
		Low	High		
Gender				0.038	0.846
Male	48	24 (50.0%)	24 (50.0%)		
Female	19	10 (52.6%)	9 (47.4%)		
Age (years)				0.726	0.394
≤60	42	23 (54.8%)	19 (45.2%)		
>60	25	11 (44.0%)	14 (56.0%)		
Histological grade				4.632	**0.031**
I/II	57	32 (56.1%)	25 (43.9%)		
III	9	1 (11.1%)	8 (88.9%)		
unknown	1				
Tumor invasion depth				0.111	0.739
T1- T2	15	9 (60.0%)	6 (40.0%)		
T3-T4	40	22 (55.0%)	18 (45.0%)		
unknown	12				
Lymph node metastasis				1.018	0.313
None	33	18 (54.5%)	15 (45.5%)		
Yes	31	13 (41.9%)	18 (58.1%)		
unknown	3				
M status				0.000	1.000
M0	62	31 (50.0%)	31 (50.0%)		
M1	5	3 (60.0%)	2 (40.0%)		
TNM stage				1.480	0.224
I/II	27	16 (59.3%)	11 (40.7%)		
III/IV	28	12 (42.9%)	16 (57.1%)		
unknown	12				

Abbreviations: PPFIA1, PTPRF interacting protein alpha 1; ESCC, esophageal squamous cell carcinoma; TNM, tumor-node-metastasis.
