# Supplementary material for: High expression of PPFIA1 in human esophageal squamous cell carcinoma correlates with tumor metastasis and poor prognosis
Source: BMC Cancer. 2023 May 9;23:417. doi: 10.1186/s12885-023-10872-9 (PMC10169376; doi:10.1186/s12885-023-10872-9)
Supplement: Supplementary file 3 — Additional file 3: Supplementary Fig. 1. The correlations between PPFIA1 expression and the prognoses of patients with malignancies were analyzed using Kaplan-Meier Plotter. [file 12885_2023_10872_MOESM3_ESM.pdf]

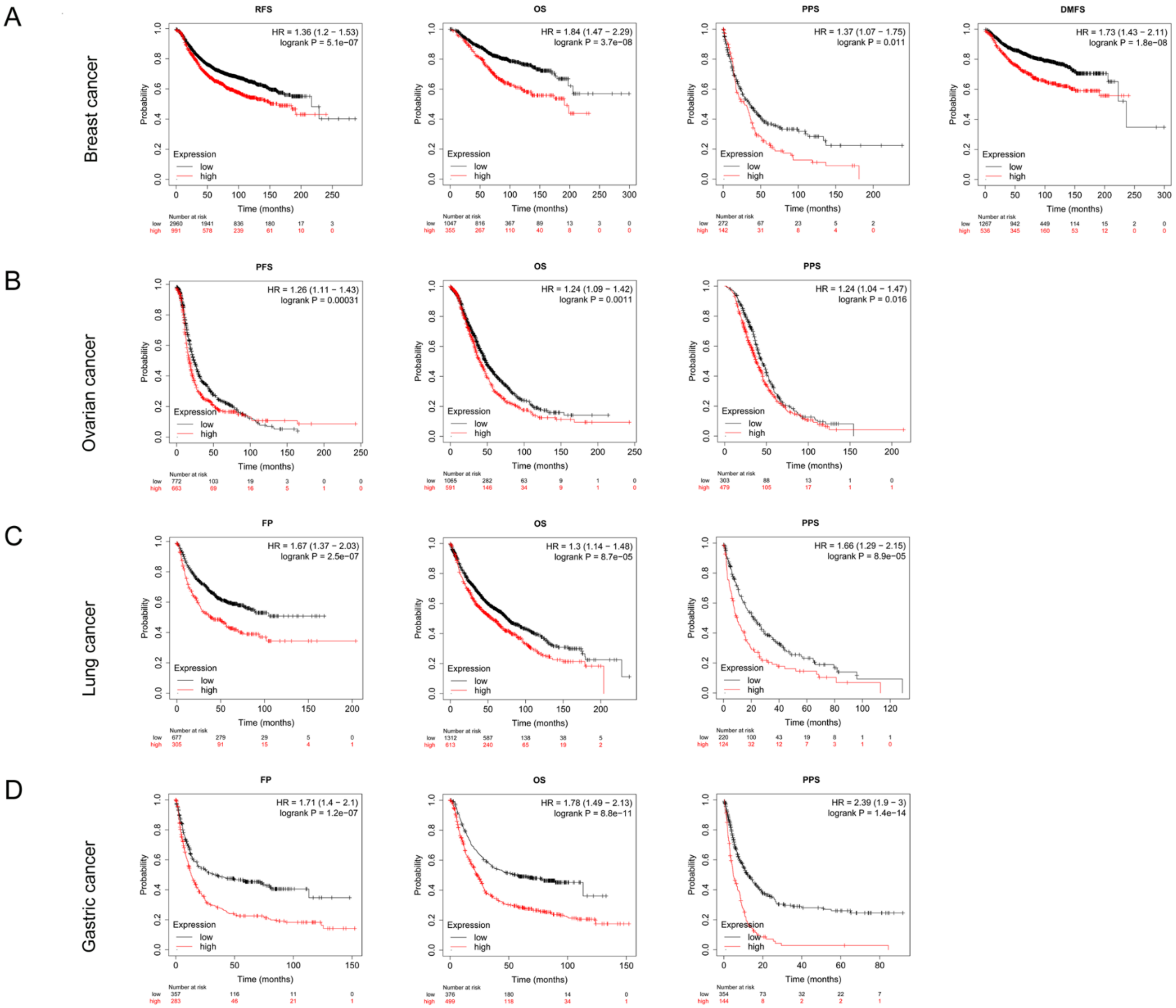
A
Breast cancer
RFS
HR = 1.36 (1.2 − 1.53)
logrank P = 5.1e−07
OS
HR = 1.84 (1.47 − 2.29)
logrank P = 3.7e−08
PPS
HR = 1.37 (1.07 − 1.75)
logrank P = 0.011
DMFS
HR = 1.73 (1.43 − 2.11)
logrank P = 1.8e−08
B
Ovarian cancer
PFS
HR = 1.26 (1.11 − 1.43)
logrank P = 0.00031
OS
HR = 1.24 (1.09 − 1.42)
logrank P = 0.0011
PPS
HR = 1.24 (1.04 − 1.47)
logrank P = 0.016
C
Lung cancer
FP
HR = 1.67 (1.37 − 2.03)
logrank P = 2.5e−07
OS
HR = 1.3 (1.14 − 1.48)
logrank P = 8.7e−05
PPS
HR = 1.66 (1.29 − 2.15)
logrank P = 8.9e−05
D
Gastric cancer
FP
HR = 1.71 (1.4 − 2.1)
logrank P = 1.2e−07
OS
HR = 1.78 (1.49 − 2.13)
logrank P = 8.8e−11
PPS
HR = 2.39 (1.9 − 3)
logrank P = 1.4e−14
Probability
Time (months)
Expression
low
high
Number at risk

Supplementary Fig. 1 The correlations between PPFIA1 expression and the prognoses of patients with malignancies were analyzed using Kaplan-Meier Plotter. Correlation of PPFIA1 expression with the RFS, OS, PPS, and DMFS of breast cancer patients (A); PFS, OS, and PPS of ovarian cancer patients (B); FP, OS, and PPS of lung cancer patients (C); and FP, OS, and PPS of gastric cancer patients (D).
