# Supplementary material for: High expression of PPFIA1 in human esophageal squamous cell carcinoma correlates with tumor metastasis and poor prognosis
Source: BMC Cancer. 2023 May 9;23:417. doi: 10.1186/s12885-023-10872-9 (PMC10169376; doi:10.1186/s12885-023-10872-9)
Supplement: Supplementary file 4 — Additional file 4: Supplementary Fig. 2. The full-length images of western blots in Fig. 6B. [file 12885_2023_10872_MOESM4_ESM.pdf]

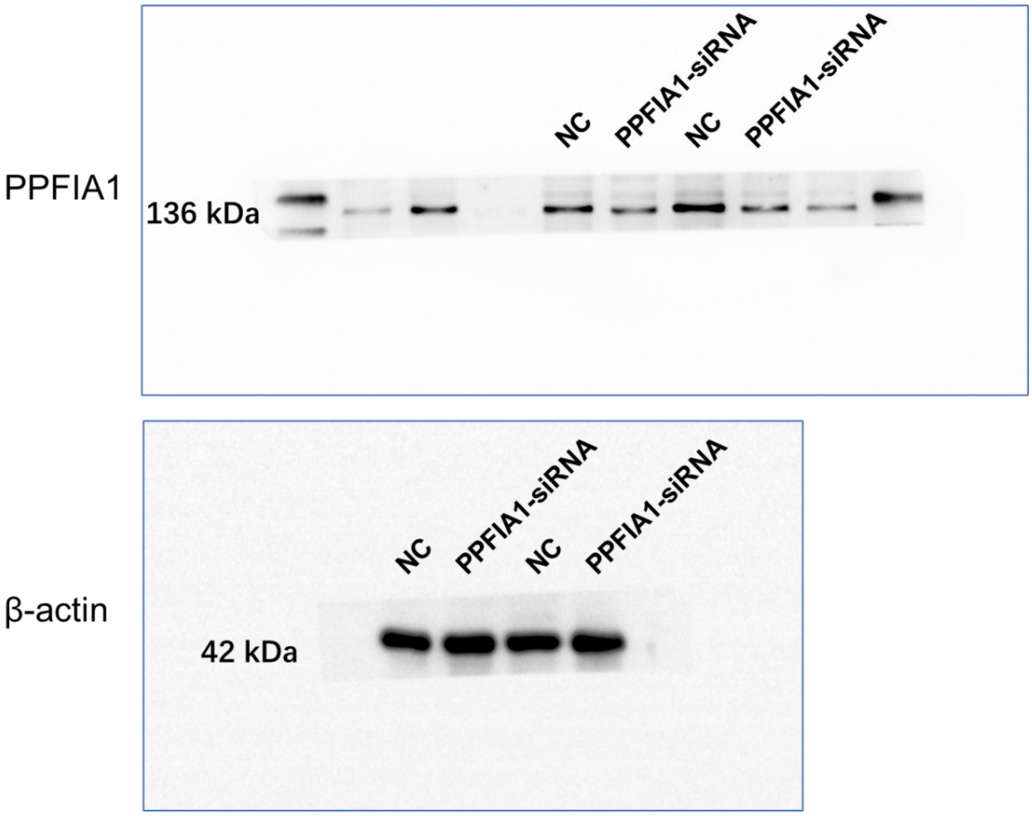


**Supplementary Fig. 2** The full-length images of western blots in Figure 6B. To avoid acquiring multiple protein bands in one field, which would interfere the results presentation of this study, protein bands which considered to be interest protein were cut prior to hybridisation with antibodies during blotting according to the hint of protein marker.
